# Quantitative metabolomics for investigating the value of polyamines in the early diagnosis and therapy of colorectal cancer

**DOI:** 10.18632/oncotarget.22885

**Published:** 2017-12-04

**Authors:** Ran Liu, Xiaohui Lin, Zuojing Li, Qing Li, Kaishun Bi

**Affiliations:** ^1^ School of Pharmacy, Shenyang Pharmaceutical University, Shenyang, 110016, China; ^2^ School of Medical Devices, Shenyang Pharmaceutical University, Shenyang, 110016, China

**Keywords:** polyamine, colorectal cancer, lasso regression analysis, quantitative metabolomics

## Abstract

As an important biomarker for cancer, polyamine levels in body fluid could be employed for monitoring the colorectal cancer (CRC), however the role of polyamines in the development and therapeutics phases of CRC remains uncertain. In this paper, the relationship between polyamines and CRC development and therapeutics had been investigated by the study of changes in plasma polyamine levels during the precancerous, developmental and treatment phases of CRC. After inducing CRC in Wistar rats by intraperitoneal injection of 1, 2-dimethylhydrazine, the animals were given a traditional Chinese medicine, Aidi injections. Firstly, the polyamine levels in the plasma of CRC, healthy and medicated rats were measured by UHPLC-MS/MS assay. In addition, Lasso regression analysis was used for screening and confirming the key markers, which can be employed for distinguishing the healthy and CRC rats as well as the CRC and medication rats. The results obtained showed that polyamine metabolism had been disrupted by CRC but returned to normal levels following Aidi injections and, in particular, putrescine and agmatine were closely correlated with CRC. Our results demonstrate the potential value of plasma polyamine metabolic profiling during the early diagnosis and medical treatment of CRC. Also, the integrated method of polyamine metabolite target analysis and lasso regression analysis can be applied in metabolomics for seeking the differential metabolites.

## INTRODUCTION

According to a report by the National Cancer Institute, colorectal cancer (CRC) is one of the most common cancers in the world. Based on five years statistical data from 2006 to 2012, CRC is the second leading cause of cancer death in the United States, and it has been estimated that over one million people are currently living with CRC in the United States [1]. In China, due to the changes in diet and lifestyle, CRC morbidity is on the rise, and it also has begun to affect the increasing number of people. Currently, the elimination of CRC is likely to depend not only on appropriate treatment but also on rapid and accurate diagnosis, and the currently available methods for Precision Medicine of CRC highlight the need to identify biomarkers for rapid diagnosis and effective treatment [2, 3].

Polyamines, aliphatic compounds possessing two or more primary or secondary amino groups, including putrescine, cadaverine, spermidine, spermine, agmatine, N-acetylputrescine, N-acetylspermine, N-acetylspermidine and 1,3-diaminopropane are metabolically derived from amino acids like L-ornithine, lysine and L-arginine. Polyamine can participate in the growth and proliferation of cells and the synthesis of proteins and nucleic acids *in vivo* [4, 5]. Polyamine homeostasis may contribute to maintain the normal physiology of active cells. Therefore, the relationship between polyamines and cancer has been extensively studied, and evidence shows that plasma and urine levels of polyamines can be used as biomarkers of cancer and also used for prognostic individual patients [6–10]. High concentrations of polyamines have been associated with rapid tumour growth [11–13], and the reduction of polyamines can inhibit cell growth [14, 15]. There is a dynamic equilibrium between polyamine biosynthesis and catabolism, and this equilibrium can be disrupted during carcinogenesis, even in the early stages of cancer development, although this disruption can be corrected by anti-cancer drugs or other therapies [16, 17]. Consequently, the systematic study of polyamine metabolic profiles may provide important information for cancer monitoring during its initial stages and allow prediction of the therapeutic effects of anti-cancer drugs. Also, as endogenous substances take part in most life activity, polyamines are more likely to be affected by traditional Chinese medicine (TCM), which can play its therapeutic effect by overall treatment with multi-target and multi-channel. Altered polyamines and their metabolic profiles could help us better understand the mechanism of TCM action and improve its effects during cancer therapy.

Based on the individual differences among experimental animals, results showing significant differences cannot be used for the analysis of disease markers. Multivariate analysis is one of the most important tools for metabolic biomarker identification in metabolomic studies and, in this study, we employed lasso regression analysis which uses dimension reduction involving regression coefficients to minimize the sum of squared residuals. This allows us to categorize and select the variables, and also eliminate the collinearity of independent variables. It also explains the variable coefficients with a weak capacity approaching zero and allows master variable for modelling. However, there is little application of Lasso regression method in the study of biomarkers. In this research, involving screening CRC biomarkers by the Lasso method, we chose the optimum correlation variables while eliminated unnecessary independent variables by the Punishment Model from the perspective of analysing independent variables (potential biomarkers) [18]. This also allowed us to retain the advantages of subset contraction to obtain a more refined dependent variable model (two-category data, cancerous or healthy individuals).

In this study, the Lasso method was initially used to analyse the UHPLC-MS/MS data acquired from the metabolomic study of polyamine metabolic profiling changes in rat plasma during CRC development and treatment, in particular to assess the role of polyamines as indicators of the efficacy of anti-cancer therapy. The results obtained in this study could be applied to anticancer drug screening and evaluation and help improve the diagnosis and treatment of CRC.

## RESULTS

### Histopathology study of CRC and treated rats

**Figure 1:**
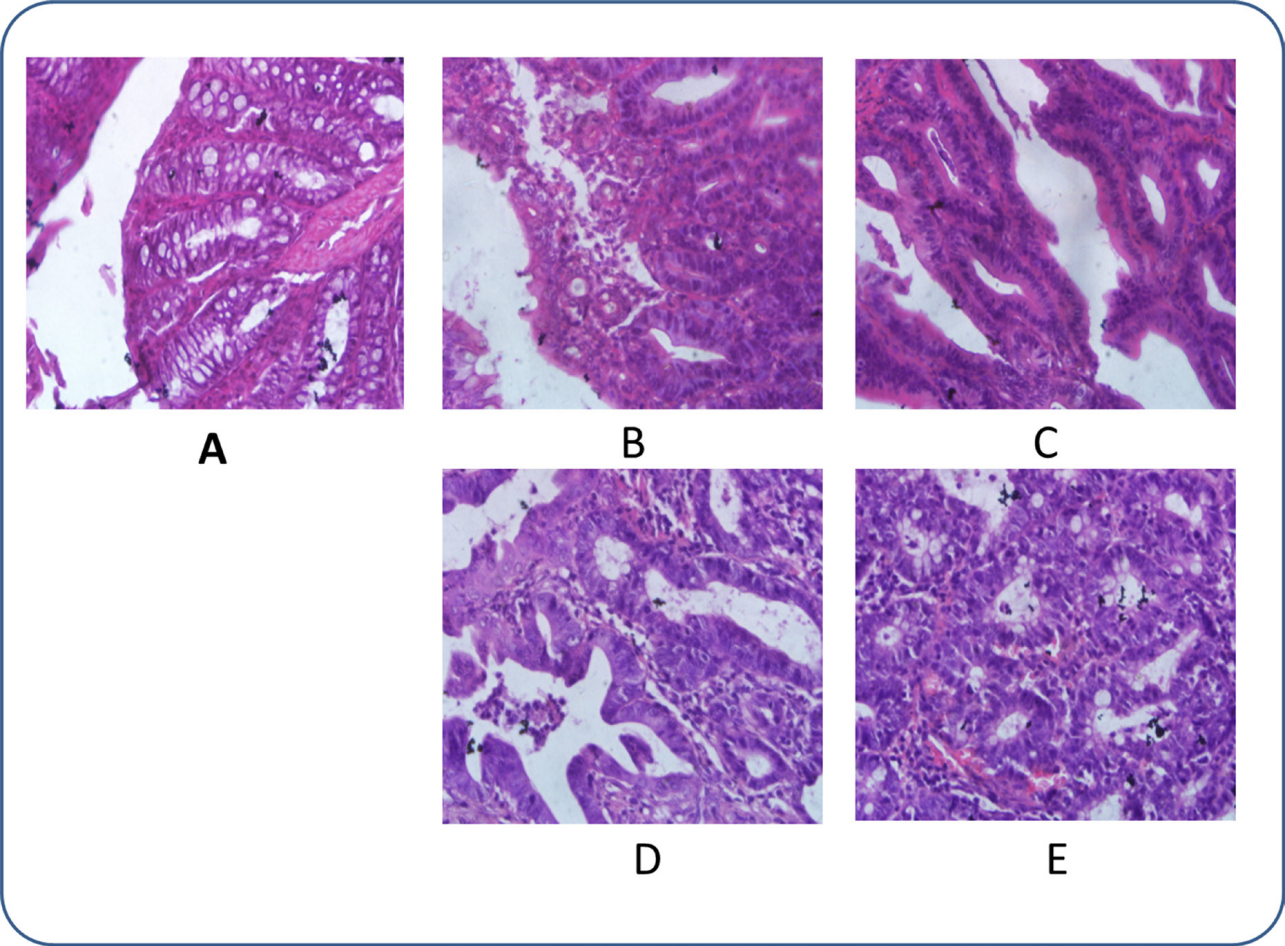
Histopathological photomicrographs of pathological rectal sections obtained after intraperitoneal injection of DMH solutions (haematoxylin and eosin (HE), × 40) (**A**) After 4 weeks of DMH treatment, nothing abnormal was detected; (**B**) After 8 weeks of DMH treatment, inflammatory cell infiltration was observed; (**C**) After 12 weeks of DMH treatment, mucosal hyperplasia was observed; (**D**) After 16 weeks of DMH treatment, rows of infiltrating neoplastic cells with marked pleomorphism were observed; and (**E**) After 20 weeks of DMH treatment, nests of adenocarcinoma were observed.

We chose 10 % (w/v) DMH (1, 2-dimethylhydrazine) saline solutions at doses of 40 mg/kg body weight per animal as our experimental carcinogenesis reagent. DMH is a commonly used chemical carcinogen with selectivity for the colorectum, and it induces a carcinogenic process similar to that of spontaneous human colorectal cancer [19–21]. The CRC group rats were induced with DMH for 20 weeks, and the histopathology of each rat rectum was examined on 4th, 8th, 12th, 16th and 20th weeks after DMH induction (Figure 1) From the histopathology of the carcinogenic process induced by DMH, it was concluded that CRC developed from mucosal hyperplasia to invasive carcinoma during our experiment. As shown in Figure 1A, the rectal sections of rats treated with DMH for 4 weeks were apparently normal. In contrast, there were inflammatory cells in the CRC rats after carcinogenesis for 8 weeks (Figure 1B). In the rats treated for 12 weeks, mucosal hyperplasia was observed in the rectal sections (Figure 1C), indicating precancerous lesions. Then, in the rats treated for 16 weeks, cells with large, dark-stained nucleoli and abnormal distributions of nucleoli (Figure 1D) were observed. At the same time, ulceration of the colorectal mucosa was also noted. The CRC rats treated with DMH for 16 weeks can be ranked as stage A according to Dukes’ classification. Finally, the CRC rats were classified as stage B after 20 weeks of DMH treatment when many adenocarcinoma nests were observed (Figure 1E) and visible tumours were observed in the mucosal layer and infiltrating into the intramuscular layer.

**Figure 2:**
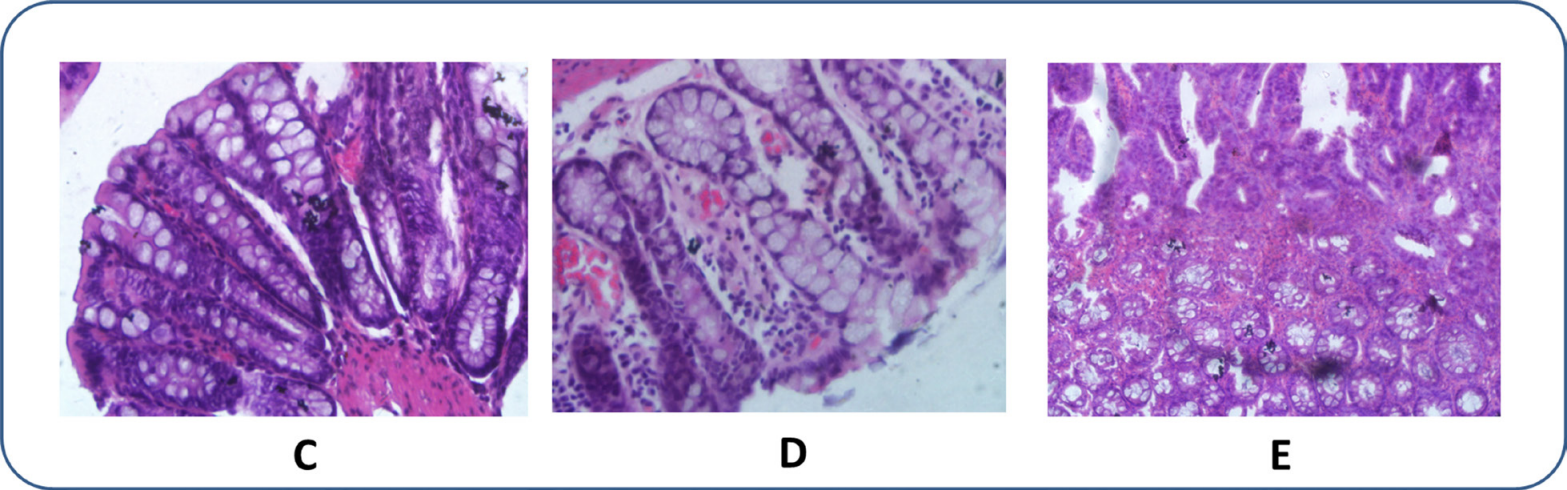
Histopathological photomicrographs of CRC rats after therapeutic treatment (HE, × 40) (**C)**: After therapy for three weeks; (**D**) After therapy for seven weeks; (**E**) After therapy for eleven weeks. Aidi injections were given at a dosage of 4 mL/kg three times a week.

Figure 2 shows rectal sections of the treated rats. Starting at the 9th week, DMH-treated rats were given anti-cancer drugs for 3 weeks. Nothing abnormal was observed in the rectal sections of these rats (Figure 2C), indicating the effectiveness of these treatments in the early stages of CRC induction. Then, at the 16th week, some inflammatory cells were observed. (Figure 2D) Finally, at the 20th week, dead cancer cells together with many inflammatory cells were observed (Figure 2E), indicating the therapeutic effect of Aidi injections.

### Polyamine metabolic profiles in CRC rats

Table 1 summarized the concentrations (mean ± SD) of plasma polyamine metabolome in CRC and normal rats. Four weeks after the first injection of DMH, there were no significant differences in polyamine levels between the CRC and control rats except for the increased level of putrescine and decreased level of N-acetylspermine (*p* < 0.05). Eight weeks after the first injection of DMH, the concentrations of several polyamines were significantly increased, including putrescine, spermine and γ-aminobutyric acid. At the twelfth week of carcinogenesis, aberrant crypt foci (ACF), which can be considered precancerous lesions of CRC, were found in the rectal tissues of the CRC rats. Compared with control rats, the levels of several polyamines, including agmatine and γ-aminobutyric acid, were significantly increased in CRC rats. Then, after the twelve weeks, abundant polyamines, including 1, 3-diaminopropane, putrescine, cadaverine, N-acetylspermidine, spermine, N-acetylspermine and agmatine, were observed in plasma, and their levels increased in parallel with the development of CRC. It has been shown that increased polyamine synthesis is a typical feature of CRC progression.

**Table 1:** Amounts of polyamine metabolome in plasma (ng/mL) from Normal Rats (*n* = 6) and CRC Rats (*n* = 6) from 4th week to 20th week in the experiment

	4^th^ week		8^th^ week		12^th^ week		16^th^ week		20^th^ week	
	Normal Rats	CRC Rats	Normal Rats	CRC Rats	Normal Rats	CRC Rats	Normal Rats	CRC Rats	Normal Rats	CRC Rats
1, 3-diaminopropane	485.7 ± 56.4	449.6 ± 111.1	734.3 ± 146.5	603.4 ± 118.6	492.1 ± 92.1	431.9 ± 5.8	229.5 ± 48.9	332.9 ± 85.4	294.4 ± 194.5	1209 ± 248^**^
putrescine	1457 ± 345	1985 ± 363^*^	1514 ± 263	2245 ± 241^**^	1173 ± 344	631.7 ± 157.1^*^	1108 ± 144	1713 ± 135^**^	830 ± 368	3832 ± 609^**^
cadaverine	205.3 ± 62.8	246.0 ± 88.6	303.9 ± 129.2	276.7 ± 69.4	220.3 ± 42.3	256.4 ± 65.3	229.1 ± 30.7	427.3 ± 139.4	171.8 ± 44.0	2674 ± 1519^*^
spermine	171.4 ± 56.1	136.5 ± 127.8	243.3 ± 101.3	422.9 ± 90.7^*^	221.2 ± 44.5	101.7 ± 58.4^*^	58.20 ± 26.07	342.2 ± 214.0^*^	52.89 ± 20.68	161.5 ± 62.9^*^
spermidine	32.34 ± 8.03	32.22 ± 21.54	23.77 ± 7.30	30.32 ± 2.47	109.0 ± 211.6	12.05 ± 6.11	77.86 ± 98.97	11.90 ± 5.63	4.74 ± 0.95	12.26 ± 7.83
N-acetylputrescine	0.10 ± 0.03	0.12 ± 0.08	0.12 ± 0.04	0.13 ± 0.01	0.11 ± 0.07	0.07 ± 0.04	0.13 ± 0.03	0.13 ± 0.08	0.09 ± 0.03	0.13 ± 0.03
N-acetylspermine	0.24 ± 0.15	0.06 ± 0.02^*^	0.48 ± 0.35	0.24 ± 0.15	0.10 ± 0.08	0.06 ± 0.02	0.03 ± 0.01	0.10 ± 0.04^**^	0.05 ± 0.01	0.21 ± 0.08^*^
N-acetylspermidine	2652 ± 994	3057 ± 663	1974 ± 1288	2317 ± 712	3126 ± 613	4663 ± 2327	3715 ± 749	3328 ± 531	2819 ± 398	3951 ± 541^*^
γ-aminobutyric acid	1247 ± 148	1232 ± 415	1254 ± 98	1518 ± 175^*^	1402 ± 58	1910 ± 376^*^	1071 ± 183	1183 ± 233	895 ± 52	997 ± 245
agmatine	625.4 ± 528.7	827 ± 671	385.7 ± 248.0	450.4 ± 205.8	354.8 ± 111.5	1326 ± 86^*^	270.7 ± 14.3	365.1 ± 61.7^**^	259.6 ± 30.4	569 ± 101^**^
L-arginine	1.508 × 104 ± 0.262 × 104	1.205 × 104 ± 0.682 × 104	1.366 × 104 ± 0.3365 × 104	1.397 × 104 ± 0.339 × 104	1.740 × 104 ± 0.843 × 104	1.431 × 104 ± 0.199 × 104^**^	1.159 × 104 ± 0.267 × 104	1.221 × 104 ± 0.789 × 104	1.262 × 104 ± 0.866 × 104	0.845 × 104 ± 0.159 × 104^*^
lysine	3633 ± 860	3262 ± 747	3449 ± 860	3679 ± 1344	4009 ± 699	3355 ± 714	2815 ± 670	3079 ± 921	2623 ± 482	2671 ± 868
L-ornithine	1600 ± 629	1645 ± 595	1467 ± 278	1902 ± 858	1045 ± 232	1586 ± 824	814 ± 225	1571 ± 849	767.9 ± 430.9	1322 ± 297
S-adenosyl-L-methionine	128.3 ± 38.7	151.1 ± 48.3	71.15 ± 63.56	155.3 ± 48.4	103.8 ± 73.8	39.12 ± 29.13	119.4 ± 26.8	98.5 ± 52.0	60.90 ± 6.55	81.0 ± 46.9

^*^p < 0.05, compared to Normal Rats.

^**^p < 0.01, compared to Normal Rats. (mean ± SD)

### Polyamine metabolic profiles in treated rats

As indicated in Table 2, the concentrations (mean ± SD) of plasma polyamine metabolome in untreated CRC rats and CRC rats receiving anti-cancer therapy were significantly different. Twelve weeks after the DMH carcinogenesis began, when the rats had been receiving anti-cancer drugs for three weeks, the levels of putrescine and agmatine were significantly reduced, and the inflammation observed on histopathological analysis had decreased in rats receiving Aidi injections.

**Table 2:** Amounts of polyamine metabolome in plasma (ng/mL) from CRC Rats(*n* = 6) and Aidi injection medication rats (*n* = 6) from 12^th^ week to 20^th^ week in the experiment

	12^th^ week		16^th^ week		20^th^ week	
	CRC Rats	Aidi^a^ Rats	CRC Rats	Aidi^b^ Rats	CRC Rats	Aidi^b^ Rats
1, 3-diaminopropane	431.9 ± 5.8	679.3 ± 274.9	332.9 ± 85.4	304.3 ± 144.0	1209 ± 248	369.1 ± 179.3^**^
putrescine	631.7 ± 157.1	1588 ± 521^*^	1713 ± 135	934 ± 186^*^	3832 ± 609	347.4 ± 155.1^**^
cadaverine	256.4 ± 65.3	176.8 ± 26.39	427.3 ± 139.4	138.7 ± 27.1^*^	2674 ± 1519	144.0 ± 45.2^*^
spermine	101.7 ± 58.4	62.68 ± 16.57	342.2 ± 214.0	77.42 ± 59.60	161.5 ± 62.9	47.35 ± 26.79^*^
spermidine	12.05 ± 6.11	169.5 ± 114.4^*^	11.90 ± 5.63	28.07 ± 20.43	12.26 ± 7.83	20.34 ± 11.76
N-acetylputrescine	0.07 ± 0.04	0.09 ± 0.02	0.13 ± 0.08	0.09 ± 0.05	0.13 ± 0.03	0.06 ± 0.04^*^
N-acetylspermine	0.06 ± 0.02	0.09 ± 0.01	0.10 ± 0.04	0.07 ± 0.04	0.21 ± 0.08	0.04 ± 0.01^**^
N-acetylspermidine	4663 ± 2327	2944 ± 1224	3328 ± 531	3708 ± 861	3951 ± 541	3881 ± 493
γ-aminobutyric acid	1910 ± 376	1517 ± 267	1183 ± 233	1055 ± 442	997 ± 245	847 ± 197
agmatine	626.1 ± 86.6	427.5 ± 177.4^*^	365.1 ± 61.7	283.1 ± 59.39^*^	569.1 ± 101.2	291.16 ± 80.35^*^
L-arginine	1.431 × 104 ± 0.199 × 104	1.369 × 104 ± 0.103 × 104	1.221 × 104 ± 0.789 × 104	0.966 × 104 ± 0.359 × 104	0.845 × 104 ± 0.159 × 104	1.003 × 104 ± 0.187 × 104
lysine	3355 ± 714	3107 ± 296	3079 ± 921	2708 ± 867	2671 ± 868	2279 ± 319
L-ornithine	1586 ± 824	1285 ± 334	1571 ± 849	1241 ± 533	1322 ± 297	917 ± 302
S-adenosyl-L-methionine	39.12 ± 29.13	79.48 ± 25.91	98.5 ± 52.0	157.0 ± 26.2	81.0 ± 46.9	97.7 ± 73.4

(mean ± SD)

Then, approximately seven weeks after the start of anti-carcinogen therapy, changes in the plasma polyamine levels were observed in the rats receiving Aidi injections and the levels of agmatine, putrescine and cadaverine were considerably reduced. After the anti-carcinogens had been given for approximately eleven weeks, the majority of the polyamine levels had returned to approximately normal levels.

### Data analysis: the lasso regression analysis

**Figure 3:**
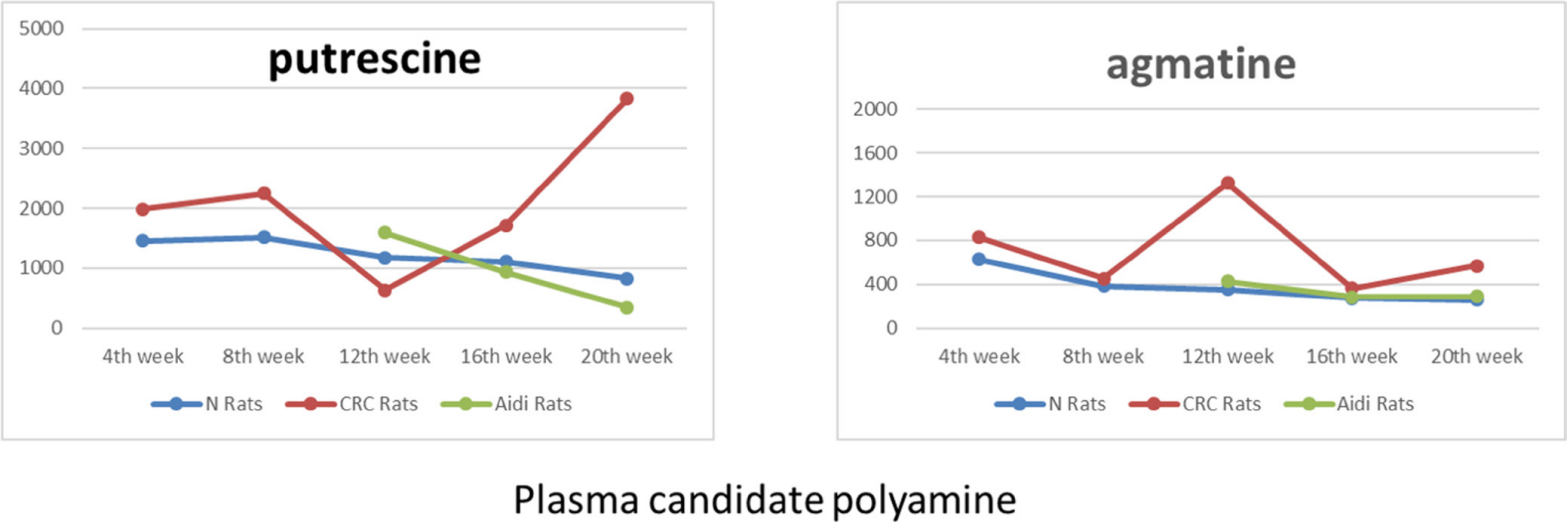
The changes in candidate polyamine values during CRC and its therapy

Prior to in-depth data analysis, data from thirty normal, thirty CRC and eighteen treated rats were randomly assigned to the train group or the test group. The raw data from chromatographic analysis of samples was processed by logarithmic transformation, to meet the needs of data analysis. Plasma polyamine metabolome raw data from twenty-six healthy rats and twenty-six CRC rats were subjected to lasso regression analysis by the R procedure and then the data were normalized and subjected to collinear index analysis. According to the distribution proportion, the variables with a distribution proportion above 50% were considered to be collinearity variables. Then, the collinearity variables of GABA, LYS, ORN and SPM were rejected. The data of twenty-six rats from each group were used as the train group, and we produced the statistic model of Logit(p) = –39.17 –0.876^*^log AGM –2.905^*^log ARG + 1.687^*^log CAD + 2.066^*^log DAP –0.5246^*^log NPUT + 2.056^*^log NSPD – 1.087^*^log NSPM + 3.675^*^log PUT – 0.2585^*^log SAM – 0.7323^*^log SPD. For testing the accuracy of the model, the data of last four rats from each group were used as test group, and the probability of 0.5 was the criterion. As shown in Figure 3, the model could distinguish between the normal and CRC rats of the test group, with an accuracy which could reach 100% and the partition degree was clear.

## DISCUSSION

### Expounding polyamine metabolism of colorectal cancer

Four weeks after the first injection of DMH, the activity of ornithine decarboxylase (ODC) and ornithine decarboxylase antizyme 1 (OAZ1) were significantly increased in CRC rats compared with normal rats. ODC is the first rate-limiting enzyme in the synthesis of polyamines, and it catalyses the conversion of L-ornithine into putrescine. A high level of ODC activity had been observed in the initial stage of colorectal cancer, and OAZ1, a type of tumour suppressor, can bind to ODC and facilitate its degradation. OAZ1 is also present at high levels in the initial stages of cancer and can inhibit high levels of polyamine formation. In our study, decreased levels of N-acetylspermine were present in plasma of CRC rats. At the same time, low activity of spermidine/spermine N1-acetyltransferase (SSAT), which can inhibit the formation of N-acetylspermine, were found. Hence, it can be concluded that abnormal polyamine metabolism can be observed during the early stages of colorectal inflammation.

Eight weeks after the first injection of DMH, the activity of polyamine metabolism-related enzymes, including ODC, OAZ1, DAO (diamine oxidase, which catalyses the biotransformation of putrescine into γ-aminobutyric acid), ADC (arginine decarboxylase, which catalyses the biotransformation of arginine into agmatine) and PAO (polyamine oxidase, one of the key enzymes in catalytic polyamine degradation) were significantly increased in CRC rats compared with normal rats. The proliferation and metabolic disruption of polyamine equilibria may begin during the inflammatory period.

At the twelfth week of carcinogenesis, agmatine were significantly increased in CRC rats. Agmatine is a known intermediate in mammalian polyamine metabolism and may play a role in cell proliferation similar to other polyamines [22]. Agmatine may be of special interest during the earliest stages of CRC. However, in the stages with ACF, the plasma levels of putrescine and spermine were significantly reduced in CRC rats compared with healthy rats even though their plasma levels had increased during the early stages of CRC. Simultaneously, according to the results of the enzyme assays, we found that the high levels of DAO activity and low levels of ODC and SSAT activity. It may be that the degradation of the polyamines exceeded their production during the later stages. We interpreted these results as showing that the low level of ODC activity and the low level of polyamines in the early stages of CRC were related to the damaged intestinal mucosa, the necrotic rectal tissue and the low numbers of neoplastic cells in the rectal sections [23, 24]. Further studies of this phenomenon are necessary and are currently being performed in our laboratory. Thus, elevated levels of agmatine and γ-aminobutyric acid as well as reduced levels of putrescine and spermine in plasma can be used as biomarkers of the early stages of CRC. Furthermore, the results suggest the importance of simultaneously determining the agmatine, γ-aminobutyric acid, putrescine and spermine in plasma for the early screening of CRC.

Then, after the 12th week, the activity levels of the enzymes related to polyamine metabolism were significantly increased, including ODC, ADC, DAO, PAO, SRM (spermidine synthase, which catalyses the biotransformation of putrescine into spermidine) and LYD (lysine decarboxylase, which catalyses the biotransformation of lysine into cadaverine). It also has been shown that polyamine synthesis is according with the CRC progression. The amounts of enzymatic activity had been shown in Supplementary Table 1.

### Investigation of the value of polyamines in the therapy of colorectal cancer

For many ineffective therapies on cancer with single anti-cancer agents, Compound Traditional Chinese Medicine with the application of compatibility from multiple active ingredients with multiple specific targets can achieve a synergistic effect. Aidi injection (ADI) is a clinical compound prescription containing Mylabris, Ginseng, Astragalus and Acanthopanax, which can inhibit growth of tumors, induce apoptosis and improve life quality of the patients. As indicated in Table 2, from the changes of polyamine metabolism, it can be concluded that the metabolism of agmatine to putrescine could be interfered by Aidi injections and this pathway could be a target of anti-cancer drug action. Simultaneously, there were low levels of activity of ODC, ADC, SRM, SMS (spermine synthase, which catalyses the biotransformation of spermidine into spermine) and LYD as well as high levels of activity of OAZ1 and SAMDC (adenosyl methionine decarboxylase, a critical enzyme in the polyamine biosynthetic pathway that generates the active pyruvoyl group for the decarboxylation of acetyl-polyamines) in rats receiving therapy compared with control CRC rats. The results of the combination of the analyses of polyamine metabolism and enzymatic activities showed that traditional Chinese medicine can alter polyamine metabolic profiles, exhaust intracellular polyamines and inhibit the proliferation of tumour cells. In addition, the phenomenon of altered polyamines and polyamine metabolic pathways confirms the benefits of traditional Chinese medicine (TCM) with multi-target and multi-coordinated effects on cancer treatment.

In conclusion, putrescine and agmatine have useful characteristics such as levels that increase with CRC progression and gradually decrease with anti-cancer therapy until they become equal to the levels of the control rats. Thus, it can be concluded that putrescine and agmatine in plasma can serve as potential target biomarkers to monitor medical treatment and to predict the regression of a cancer. Also, the pathway of agmatine to putrescine could be the target of anti-cancer drug action. The amounts of enzymatic activity had been shown in Supplementary Table 2.

### Data analysis: the lasso regression analysis

**Figure 4:**
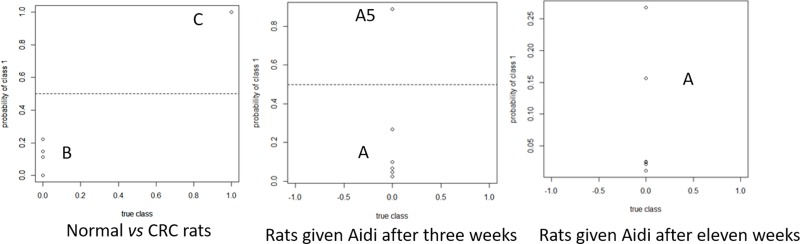
Probability distribution result by lasso analysis in normal, CRC and medicated rat plasma A1~6 for rats given Aidi after three weeks, A7~12 for rats give Aidi after eleven weeks. B19~22 for normal rats, and C19~22 for CRC rats. Variable 1 for cancerous animals and 0 for healthy animals.

Then, the established model was used to analyze the plasma polyamine level data from the rats receiving medication. Table 3 and Figure 4 show the discriminant analysis results of lasso analysis from rats receiving Aidi injections after three and eleven weeks. It had been shown that, after medication for three weeks, the discrimination results of five out of six CRC rats were below 0.5. Accordingly, it appeared that treatment with Aidi injections for 21 days returned the plasma polyamine levels to normal and, combined with the results of the histopathology study, this confirmed the therapeutic effect of Aidi injections. Then, after treatment for eleven weeks, the discrimination results of all CRC rats were below 0.5 and were close to 0. So, it appears that the level of plasma polyamine metabolism can be returned to normal by medical treatment. Combined with the histopathology results, it can be concluded that the cancer can be effectively treated by Aidi injections at its early stage. Also, the results also confirmed the role of polyamines as indicators of the efficacy of anti-cancer therapy. In addition, the Lasso regression analysis method can be applied not only to distinguish between healthy individuals and those with cancer, and also to the estimate the curative effect of anti-cancer drugs. The comparation of Lasso regression analysis and other method had been shown in Supplementary Table 4.

**Table 3:** Discriminant analysis result by lasso analysis from healthy, CRC and medication rats plasma

No	Probability value	No	Probability value
B^*^19	0.04047	C^*^19	0.999
B20	0.004938	C20	0.999
B21	0.2323	C21	0.999
B22	0.1669	C22	0.999
A^*^1	0.04687	A^*^7	0.1563
A2	0.02516	A8	0.02516
A3	0.06654	A9	0.01134
A4	0.2676	A10	0.02112
A5	0.889	A11	0.02427
A6	0.0979	A12	0.2676

^*^A1~6 for rat medication by Aidi after 21d. A7~12 for rat medication by Aidi after 77d. B19~22 for healthy rats. C19~22 for CRC rats.

*p* < 0.05, compared to CRC Rats.

^**^p < 0.01, compared to CRC Rats.

a: Aidi Rats: Aidi injection medication rats.

## MATERIALS AND METHODS

### Materials and agents

The reference standards of 1, 3-diaminopropane, putrescine, cadaverine hydrochloride, spermidine hydrochloride, spermine, agmatine sulphate salt, N-acetylputrescine hydrochloride, N-acetylspermine trihydrochloride, N-acetylspermidine dihydrochloride, L-ornithine hydrochloride, lysine, L-arginine, S-adenosyl-L-methionine, γ-aminobutyric acid and 1,6-diaminohexane (used as an internal standard) were all obtained from Sigma-Aldrich (St. Louis, MO, USA). High-performance liquid chromatography (HPLC)-grade methanol was obtained from Fisher Chemicals (Fair Lawn, NJ, USA) while heptafluorobutyric acid (HFBA) was obtained from Sigma-Aldrich (St. Louis, MO, USA). All other reagents were of analytic grade and redistilled and deionised water was used throughout the study.

DMH was obtained from Sigma-Aldrich (St. Louis, MO, USA). Aidi injections, a traditional Chinese medicine approved for clinical use, were developed in China and have been used since 2002. The Aidi injections were prepared according to the Ministerial Standards of Chinese Medicine. The Aidi injections contained Mylabris (the dried polypide of Mylabris phalerata Pall or Mylabris cichorii Linnaeus), Ginseng Radix Et Rhizoma (the dried root and rhizome of Panax ginseng C.A.Mey.), Astragali Radix (the dried root of Astragalus membranaceus (Fisch.) Bge. var. mongholicus (Bge.) Hsiao and Acanthopanacis Senticosi Radix Et Rhizoma Seu Caulis (the dried root and rhizome of Acanthopanax senticosus Harms). Aidi injections are an effective Chinese herbal preparation with anti-cancer activity for the treatment of liver and lung cancer as well as CRC. It has been reported that triterpenoid saponins, lignans, coumarins, and flavones, including ginsenoside Rg1, Rb1, Rc, Re, Rd, syringin B, syringin E, isofraxidin and cishhdryin are the most active components of Aidi injections [25]. The medicines in our experiment were prepared immediately prior to use.

### Experimental animals and sample collection

Male and female Wistar rats (220–250 g) were obtained from the Experimental Animal Centre of Shenyang Pharmaceutical University and given standard rodent chow and unlimited access to water in an air-conditioned animal centre at a temperature of 22 ± 2°C and a relative humidity of 50 ± 10 % with a natural light-dark cycle during the experimental period. The animal study was carried out following the Animal Experimentation Guidelines of Shenyang Pharmaceutical University, and the protocol was approved by the Animal Ethics Committee of the institution.

A total of 78 rats were divided into two groups, CRC rats (*n* = 48) and normal rats (*n* = 30). The CRC rats were given DMH solutions as described previously [19–20]. Briefly, the CRC rats received intraperitoneal injections of DMH solution (DMH dissolved in saline to produce 10 % solutions) at a dosage of 40 mg/kg once a week for 8 weeks. Normal rats received the same volume of vehicle without DMH at the same time and for the same period. During the carcinogenesis period, plasma and rectal samples were collected from the CRC and normal rats (*n* = 6) during the fourth and eighth weeks. Then, during the 9th week, the CRC rats (*n* = 36) were randomly divided into two groups, a CRC group (sequentially injected with DMH solution at a dosage of 40 mg/kg once a week for 3 weeks, *n* = 18) and an Aidi group (injected with Aidi at a dosage of 4 mL/kg three times a week, *n* = 18). On the 12th, 16th and 20th weeks, 24 hours after the last dose, plasma and rectal samples from each group were collected (*n* = 6 for each group) and immediately frozen at –80°C until analysis. Histopathological examination of the rectal samples was used to confirm the success of the CRC model and to observe the therapeutic effects of the treatments. There were equal numbers of male and female rats in each group throughout the experiment.

### Polyamine detection and analysis

To determine the polyamine metabolism, we used a simple and sensitive ultra HPLC (UHPLC)-tandem mass spectrometry (MS/MS) method as described in our previous paper [26]. Briefly, chromatographic separation was achieved with gradient elution using a mobile phase composed of 0.05 % HFBA in water (A) and 0.05 % HFBA in methanol (B). The 9.0 min UHPLC gradient program was as follows: 20 % B for 0.01–2.00 min; 20 % B→50 % B for 2.01–4.00 min; 50 % B for 4.01–6.00 min; and 20 % B for 6.01–9.00 min. The MS detection was carried out on a QTRAP™ 4000 MS/MS system from AB Sciex equipped with a Turbo Ion Spray source (Foster City, CA, USA). The detection of analytes was in multiple reaction monitoring mode(MRM) with electrospray positive ionization (ESI+). Each 250 μL plasma sample was obtained by adding 250 μL methanol (containing 0.1 % acetic acid), and then vortex mixing for 5 min and centrifuging for 3 min at 15,000 rpm and 4°C for twice. Then, the supernatant was transferred to another Eppendorf microtube and evaporated to dryness at 30°C under a stream of air. The residue was dissolved in 50 μL methanol containing 0.05 % HFBA and water containing 0.05 % HFBA (20:80, v/v), and 5 μL of the solution was injected for analysis.

Polyamine concentrations were obtained from the calibration curves and expressed as the mean ± standard deviation (SD). All the statistical analyses were performed using the Statistical Package for Social Sciences (SPSS) 19.0 statistical software (IBM, Chicago, IL, USA). Student's t and Mann-Whitney U tests were carried out and a *P* values < 0.05 were deemed statistically significant. The results of method validation for the analytes had been shown in Supplementary Table 3.

The activities of enzymes involved in polyamine metabolism were determined using ELISA (enzyme-linked immunosorbent assay) kits according to the manufacturer's recommendations.

### Data statistics and analysis

Lasso regression analysis was carried out based on the convergence variable set, which involves the method of shrinkage estimation. The basic variable selection hypothesis of Lasso is under the conditions that the sum from the absolute value of the regression coefficient was less than a constant, to minimize the sum of squared residuals and obtain a regression coefficient equal to zero [27, 28].

R language is widely used in statistics and this is applied to data exploration and statistical analysis. The raw data from the chromatographic analysis of samples was processed by Lasso regression analysis using R language.

The Lasso analysis procedure was as follows:

1. Data pre-processing:
  The raw data was processed by normalized transformation, and applying the programs of perturb and glmnet to examine the collinear variables, removing the variables which had a distribution ratio more than 50%.
2. Assigning the train group and test group:
  Based on the data size, according to the proportion of 7:3 or 8:2, the sample data was randomly divided into two groups while fourteen polyamine metabolites served as variables. One from the train group was used in model building and one from the test group was used for model checking.
3. Using the train group to establish the statistical model by Lasso analysis:
  Through iterative calculation, the coefficient of unnecessary variables could be reduced to zero for variable screening.
4. Using the test group to examine the accuracy of the statistical model.

## CONCLUSIONS

Studies of polyamine metabolic profiling during cancer development and therapy are rare and, here, we have presented a detailed and systematic analysis of plasma polyamine metabolic profiles in CRC. Our study demonstrated the relationship between plasma polyamine metabolite levels and cancer progression as well as cancer treatment process. Using the Lasso method, a discriminant equation was developed to distinguish between healthy rats and those with cancer. The equation accuracy can reach 100 % using a test set, which offers a clear distinction. At the same time, using the Lasso method to examine individual polyamine metabolic profiles after administration, the polyamine levels return to normal in animals given Aidi injections.

In summary, our study suggests that putrescine and agmatine are sensitive biomarkers which can be used to evaluate anti-cancer drugs, while the metabolism from agmatine to putrescine is disrupted by anti-cancer drug treatment and this pathway could be the target for anti-cancer drug action. These results suggest that determining polyamine metabolic profiles could contribute to CRC biomarker profiling and anticancer drug screening. Furthermore, a detailed and intensive study involving a large number of human subjects is currently being planned with the cooperation of a local hospital. In addition, this approach of evaluating the therapeutic effect by altered polyamine levels and metabolic pathways could be applied to study the efficacy and mechanisms of action of TCM.

## SUPPLEMENTARY MATERIALS TABLES




