# Supplementary material for: Quantitative metabolomics for investigating the value of polyamines in the early diagnosis and therapy of colorectal cancer
Source: Oncotarget. 2017 Dec 4;9(4):4583–92. doi: 10.18632/oncotarget.22885 (PMC5796997; doi:10.18632/oncotarget.22885)
Supplement: Supplementary file 1 [file oncotarget-09-4583-s001.pdf]

Quantitative metabolomics for investigating the value of polyamines in the early diagnosis and therapy of colorectal cancer

SUPPLEMENTARY MATERIALS

Supplementary Table 1: Amounts of enzymatic activity in plasma (ng/mL) from Normal Rats (n = 6) and CRC Rats (n = 6) from 4th week to 20th week in the experiment

	4th week		8th week		12th week		16th week		20th week	
	**CRC Rats**	**Normal Rats**	**CRC Rats**	**Normal Rats**	**CRC Rats**	**Normal Rats**	**CRC Rats**	**Normal Rats**	**CRC Rats**	**Normal Rats**
Ornithine Decarboxylase	1.78 ± 0.22*	0.71 ± 0.07	1.32 ± 0.14*	0.73 ± 0.11	0.51 ± 0.04*	0.87 ± 0.08	1.73 ± 0.05*	0.66 ± 0.03	1.63 ± 0.06*	0.81 ± 0.20
Arginine Decarboxylase	0.36 ± 0.08	0.46 ± 0.39	0.81 ± 0.13*	0.37 ± 0.36	0.63 ± 0.02	0.59 ± 0.15	0.81 ± 0.14*	0.28 ± 0.39	0.76 ± 0.04*	0.35 ± 0.09
S-adenosine Methionine Decarboxylase	0.57 ± 0.14	0.47 ± 0.36	0.68 ± 0.02	0.55 ± 0.11	0.48 ± 0.14	0.62 ± 0.18	0.51 ± 0.18	0.71 ± 0.04	0.64 ± 0.06	0.74 ± 0.05
Arginine Decarboxylase	0.78 ± 0.15	0.63 ± 0.11	0.78 ± 0.03	0.77 ± 0.11	0.64 ± 0.06	0.71 ± 0.07	1.39 ± 0.13*	0.64 ± 0.04	1.36 ± 0.23*	0.59 ± 0.06
Ornithine Decarboxylase Antizyme 1	1.42 ± 0.06*	0.75 ± 0.05	1.57 ± 0.74*	0.66 ± 0.05	3.92 ± 0.46*	0.74 ± 0.13	2.78 ± 0.10*	0.74 ± 0.09	2.61 ± 0.15*	0.72 ± 0.07
Polyamine Oxidase	1.26 ± 0.07	1.29 ± 0.03	1.92 ± 0.06*	1.23 ± 0.05	1.19 ± 0.07	1.20 ± 0.07	2.60 ± 0.01*	1.31 ± 0.05	2.92 ± 0.57*	1.34 ± 0.13
Spermidine and Spermine Acetyl Transferase	27.79 ± 3.69*	44.39 ± 4.40	45.50 ± 4.30	48.03 ± 8.27	33.62 ± 2.74*	59.25 ± 5.03	34.65 ± 3.61	56.78 ± 4.03	36.33 ± 5.12	50.57 ± 3.30
Spermine Synthase	3.31 ± 0.31	3.07 ± 0.58	3.19 ± 0.36	3.20 ± 0.48	3.16 ± 0.76	4.17 ± 0.60	3.02 ± 0.43	3.79 ± 0.78	2.55 ± 0.93	3.68 ± 0.31
Diamine Oxidase	12.51 ± 0.90	11.29 ± 0.79	19.51 ± 0.64*	11.73 ± 0.63	21.26 ± 1.21*	12.52 ± 2.35	22.80 ± 0.88*	11.66 ± 0.36	24.02 ± 0.33*	10.82 ± 1.38
Lysine Decarboxylase	6.69 ± 0.12	7.26 ± 0.36	6.54 ± 0.09	6.70 ± 0.35	6.08 ± 0.47	7.34 ± 0.19	10.99 ± 0.61*	7.39 ± 0.43	12.10 ± 0.16*	6.36 ± 0.17

*$p < 0.05$, compared to Normal Rats.
(mean ± SD)

Supplementary Table 2: Amounts of enzymatic activity in plasma (ng/mL) from Aidi injection medication rats ($n = 6$) from 12th week to 20th week in the experiment

	12th week		16th week		20th week	
	**CRC rats**	**Aidi rats**	**CRC rats**	**Aidi rats**	**CRC rats**	**Aidi rats**
Ornithine Decarboxylase	0.51 ± 0.04	0.75 ± 0.14*	1.73 ± 0.05	0.37 ± 0.37*	1.63 ± 0.06	0.72 ± 0.19*
Arginine Decarboxylase	0.63 ± 0.02	0.23 ± 0.27*	0.81 ± 0.14	0.35 ± 0.09*	0.76 ± 0.04	0.30 ± 0.07*
S-adenosine Methionine Decarboxylase	0.48 ± 0.14	0.19 ± 2.24	0.51 ± 0.18	8.70 ± 0.68*	0.64 ± 0.06	0.95 ± 0.45
Arginine Decarboxylase	0.64 ± 0.06	0.02 ± 0.19*	1.39 ± 0.13	1.23 ± 0.15	1.36 ± 0.23	0.31 ± 0.45*
Ornithine Decarboxylase Antizyme 1	3.92 ± 0.46	1.70 ± 0.13*	2.78 ± 0.10	2.58 ± 0.25	2.61 ± 0.15	4.32 ± 0.08*
Polyamine Oxidase	1.19 ± 0.07	1.94 ± 0.07	2.60 ± 0.01	1.80 ± 0.01*	2.92 ± 0.57	2.02 ± 0.18
Spermidine and Spermine Acetyl Transferase	33.62 ± 2.74	44.49 ± 0.44	34.65 ± 3.61	51.73 ± 6.85*	36.33 ± 5.12	47.82 ± 2.06
Spermine Synthase	3.16 ± 0.76	2.42 ± 0.69	3.02 ± 0.43	1.94 ± 0.12*	2.55 ± 0.93	2.69 ± 0.45
Diamine Oxidase	21.26 ± 1.21	18.60 ± 0.55	22.80 ± 0.88	22.18 ± 0.79	24.02 ± 0.33	13.69 ± 1.27
Lysine Decarboxylase	6.08 ± 0.47	6.01 ± 0.12	10.99 ± 0.61	5.90 ± 0.67*	12.10 ± 0.16	5.44 ± 0.74*

$^{*}p < 0.05$, compared to CRC Rats.
(mean ± SD)

Supplementary Table 3: The calibration validation for the analytes in plasma. See Supplementary_Table_3

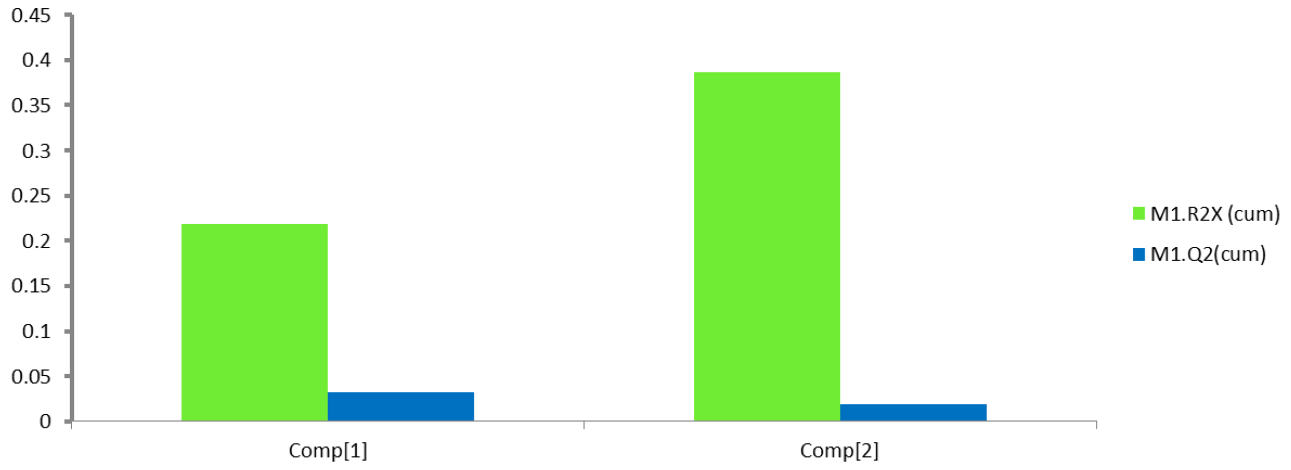

Supplementary Table 4-1: Analysis of the cumulative contribution rate of polyamine data by PCA in normal and CRC rat's plasma.

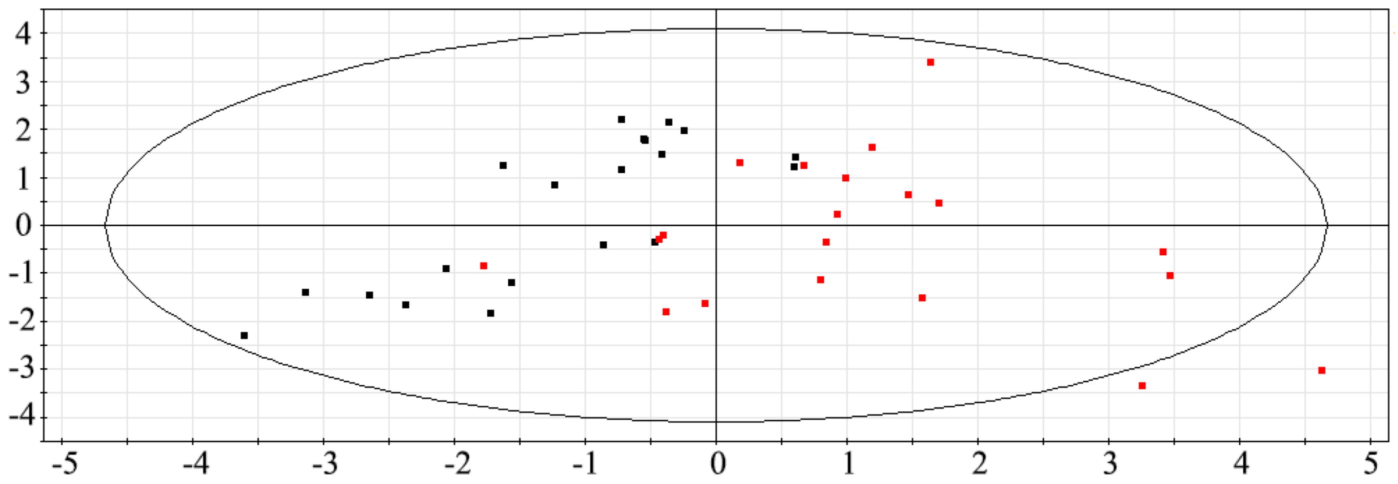

Supplementary Table 4-2: Principal component oval score of the data from health and CRC rat's plasma by PCA analysis.

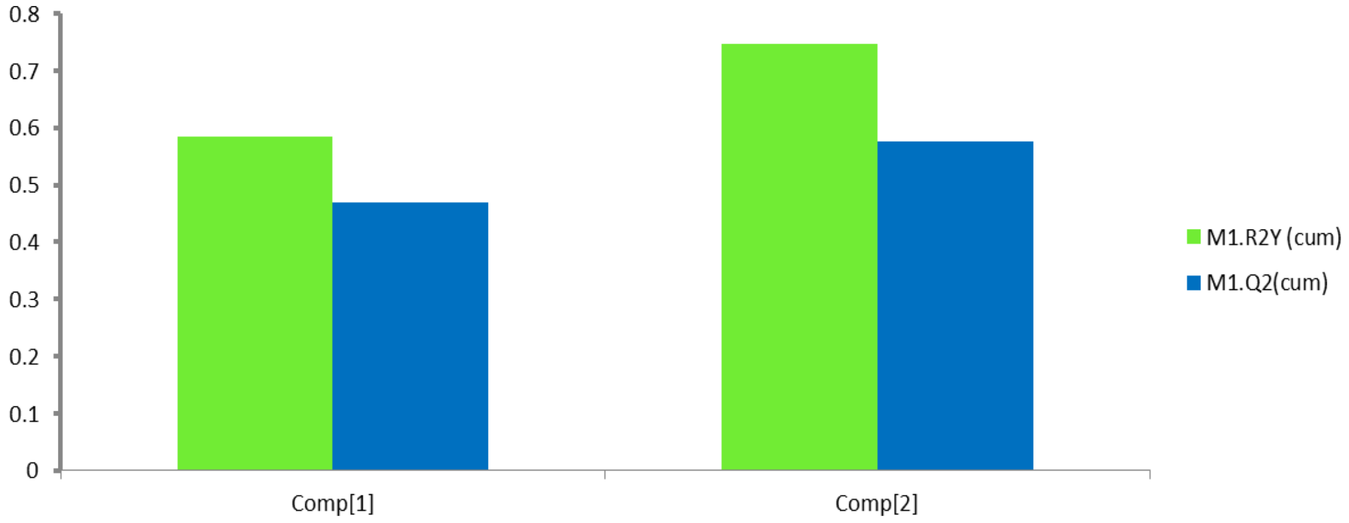

Supplementary Table 4-3: Analysis of the cumulative contribution rate of the main components of the data by PLS-DA in normal and CRC rats.

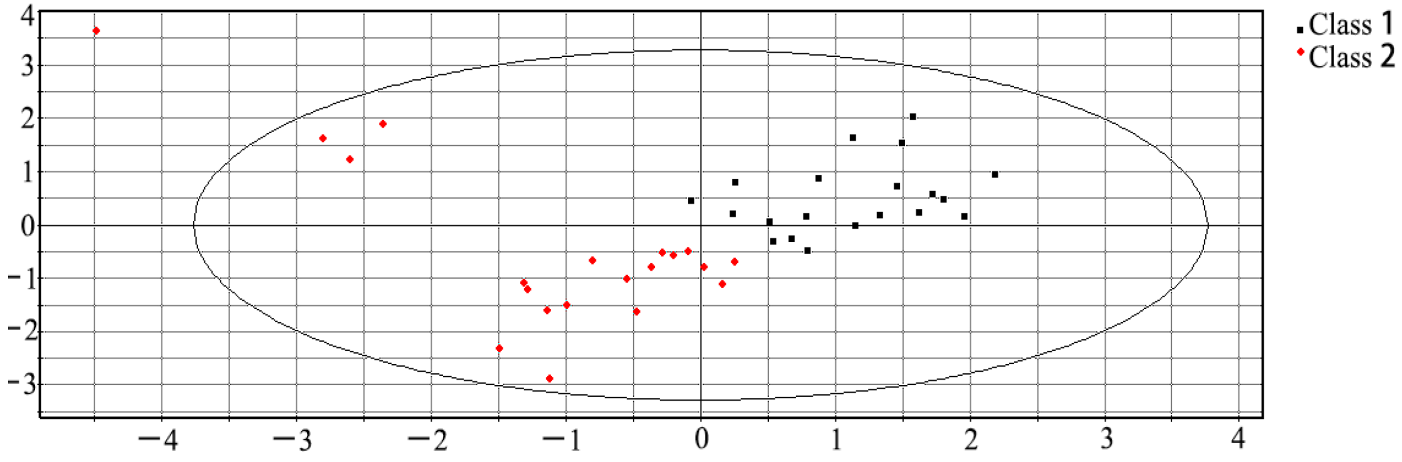

Supplementary Table 4-4: Principal component oval score of the data from normal and CRC rats's plasma by PLS-DA analysis.

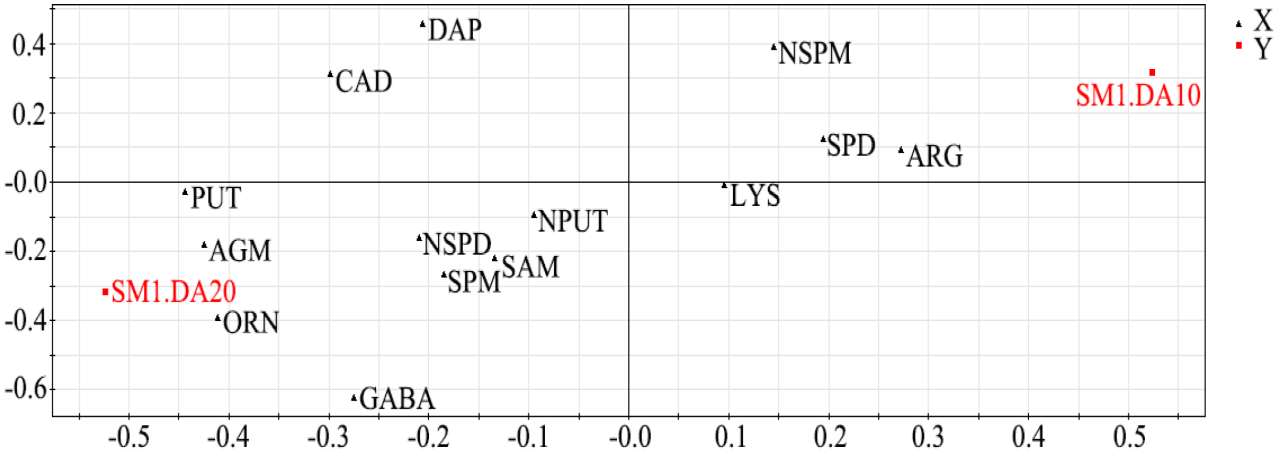

Supplementary Table 4-5: Load chart of the data from health and CRC rat's plasma by PLS-DA analysis.

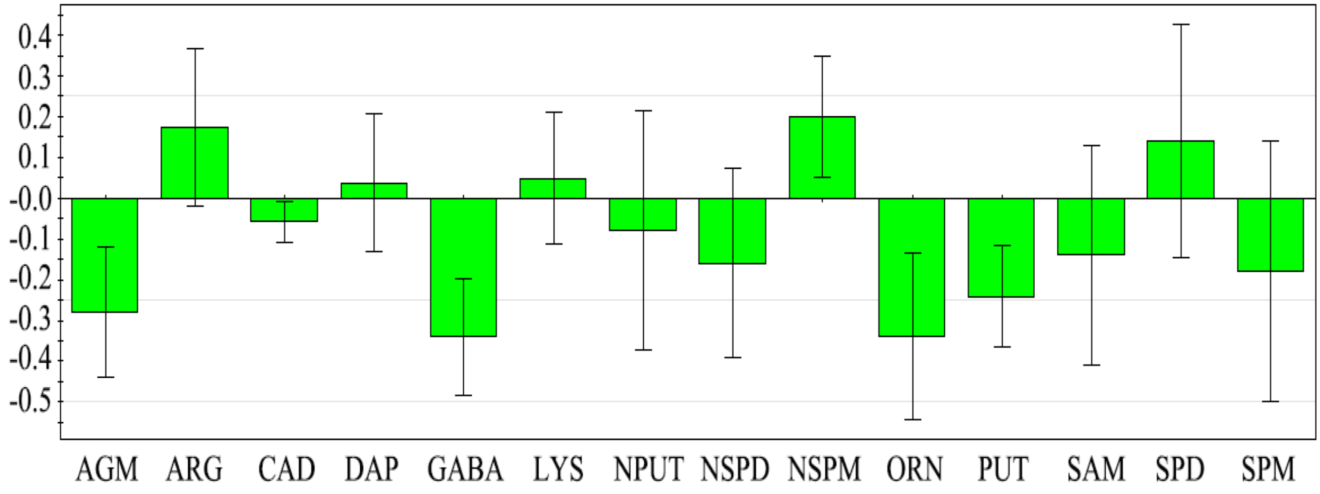

Supplementary Table 4-6: Principal component coefficient of the data from health and CRC rat's plasma by PLS-DA analysis.

Supplementary Table 4-7: Principal component coefficient factor of the data from health and CRC rat's plasma by PLS-DA analysis

Var ID(Primary)	M1.CoeffC S[2]($M1.DA10)	Var ID(Primary)	M1.CoeffC S[2] ($M1.DA10)
$constant	0.9874	NSPD	–0.1604
AGM	–0.2797	NSPM	0.1998
ARG	0.1724	ORN	–0.3386
CAD	–0.0575	PUT	–0.2414
DAP	0.0306	SAM	–0.1399
GABA	–0.3407	SPD	0.141
LYS	0.04827	SPM	–0.1804
NPUT	–0.07964		

Supplementary Table 4-8: The iteration data by logistic regression analysis from normal and CRC rat plasma

step	–2 log likelihood	Cox & Snell R^2	Nagelkerke R^2
1	48.76[a]	0.243	0.324
2	40.32[b]	0.375	0.500
3	32.99[b]	0.471	0.628
4	18.10[c]	0.623	0.830

a. Because the parameter estimates are less than0.001, the number of iterations is estimated at 5 iterations.
b. Because the parameter estimates are less than.001, the number of iterations is estimated at 6 iterations.
c. Because the parameter estimates are less than.001, the number of iterations is estimated at 9 iterations.

Supplementary Table 4-9: Classification by logistic regression analysis from normal and CRC rat plasma

observed			forecast		Percentage correction
			VAR00016		
			0.00	1.00	
step 1	VAR00016	0.00	18	4	81.8
		1.00	5	17	77.3
	Total percentage				79.5
step 2	VAR00016	0.00	16	6	72.7
		1.00	5	17	77.3
	Total percentage				75.0
step 3	VAR00016	0.00	17	5	77.3
		1.00	4	18	81.8
	Total percentage				79.5
step 4	VAR00016	0.00	21	1	95.5
		1.00	2	20	90.9
	Total percentage				93.2

Cut value 0.500

Supplementary Table 4-10: Variable analysis by logistic regression analysis from normal and CRC rat plasma

		B	S.E,	Wals	df	Sig.	Exp (B)
step 1[a]	PUT	0.00177	0.00067	6.980	1	0.008	1.002
	constant	–2.783	1.065	6.826	1	0.009	0.062
step 2[b]	AGM	0.223	0.0981	5.177	1	0.023	1.250
	PUT	0.00213	0.00081	6.849	1	0.009	1.002
	constant	–4.941	1.640	9.07	1	0.003	0.007
step 3[c]	AGM	0.2331	0.1132	4.242	1	0.039	1.263
	constant	0.00375	0.00173	4.711	1	0.030	1.004
	PUT	0.00279	0.00109	6.551	1	0.010	1.003
	constant	–11.055	3.582	9.52	1	0.002	0.000
step 4[d]	AGM	0.4866	0.2302	4.469	1	0.035	1.627
	ARG	–0.00092	0.00037	6.178	1	0.013	0.999
	GABA	0.00916	0.00487	3.534	1	0.060	1.009
	PUT	0.00631	0.00230	7.520	1	0.006	1.006
	constant	–12.50	6.629	3.559	1	0.059	0.000

a. Variables entered in step 1: PUT
b. Variables entered in step 2: AGM
c. Variables entered in step 3: GABA
d. Variables entered in step 4: ARG
