# Supplementary material for: Quantitative metabolomics for investigating the value of polyamines in the early diagnosis and therapy of colorectal cancer
Source: Oncotarget. 2017 Dec 4;9(4):4583–92. doi: 10.18632/oncotarget.22885 (PMC5796997; doi:10.18632/oncotarget.22885)
Supplement: Supplementary file 2 [file oncotarget-09-4583-s002.doc]

Supplementary Table 3. The calibration validation for the analytes in plasma.

| Analyte | LOQ (ng/ml) | Liner range (ng/ml) | Slope | Intercept | Regression coefficient |
| --- | --- | --- | --- | --- | --- |
| 1,3-diaminopropane | 15.00 | 15.00 – 1500 | 5.851×10^-4^ | 3.336×10^-2^ | 0.9903 |
| putrescine | 50.00 | 50.00 – 5000 | 6.778×10^-2^ | 0.4565 | 0.9908 |
| cadaverine | 50.00 | 50.00 – 5000 | 4.886×10^-3^ | 1.300×10^-2^ | 0.9918 |
| spermidine | 200.0 | 200.0 – 2.000×10^4^ | 23.39 | -2.356 | 0.9908 |
| spermine | 200.0 | 200.0 –2.000×10^4^ | 6.901 | -2.163 | 0.9924 |
| agmatine | 2.00 | 2.00 – 200.0 | 4.690×10^-2^ | -9.61×10^-3^ | 0.9957 |
| L-ornithine | 200.0 | 200.0 – 2.000×10^4^ | 7.973 | -5.887 | 0.9976 |
| lysine | 500.0 | 500.0 –5.000×10^4^ | 2.569 | 5.885 | 0.9989 |
| L-arginine | 500.0 | 500.0 –5.000×10^4^ | 4.655 | 8.39 | 0.9991 |
| S-adenosyl-L-methionine | 2.00 | 2.00 – 200.0 | 0.1225 | 0.1216 | 0.9917 |
| N-acetylputrescine | 0.10 | 0.10 – 10.00 | 6.853×10^-2^ | 7.432×10^-2^ | 0.9983 |
| N-acetylspermine | 0.10 | 0.10 – 10.00 | 1.286×10^-2^ | 0.1213 | 0.9903 |
| N-acetylspermidine | 0.10 | 0.10 – 10.00 | 9.21×10^-3^ | 2.383×10^-3^ | 0.9929 |
| γ-aminobutyric acid | 50.00 | 50.00 – 5000 | 1.319×10^-3^ | 8.91×10^-3^ | 0.9949 |

| Analytes | Concentration  ( ng/ml ) | Intra - day  RSD% | Inter - day  RSD% | Accuracy RE% |
| --- | --- | --- | --- | --- |
| 1,3-diaminopropane | 30.00 | 13.3 | 3.7 | -12.2 |
|  | 300.0 | 11.4 | 2.8 | -10.4 |
|  | 1200 | 14.0 | 1.2 | -9.3 |
| putrescine | 100.0 | 11.1 | 1.3 | 6.5 |
|  | 1000 | 6.9 | 6.8 | 11.9 |
|  | 4000 | 3.5 | 9.8 | -6.5 |
| cadaverine | 100.0 | 6.4 | 3.9 | 8.3 |
|  | 1000 | 4.6 | 2.0 | 5.7 |
|  | 4000 | 4.4 | 9.7 | -14.3 |
| spermidine | 400.0 | 9.1 | 1.2 | 12.8 |
|  | 4000 | 6.0 | 5.5 | 8.5 |
|  | 1.600×10^4^ | 2.4 | 5.9 | -4.5 |
| spermine | 400.0 | 5.7 | 1.3 | -13.2 |
|  | 4000 | 6.3 | 11.6 | -10.2 |
|  | 1.600×10^4^ | 8.7 | 0.9 | 13.9 |
| agmatine | 4.00 | 11.2 | 6.3 | 12.5 |
|  | 40.00 | 14.6 | 1.1 | 8.7 |
|  | 160.0 | 5.3 | 8.8 | 1.6 |
| L-ornithine | 400.0 | 12.2 | 6.2 | 3.7 |
|  | 4000 | 10.5 | 5.4 | 10.6 |
|  | 1.600×10^4^ | 10.4 | 11.9 | 3.5 |
| lysine | 1000 | 7.9 | 5.0 | -1.5 |
|  | 1.000×10^4^ | 11.9 | 1.3 | -6.9 |
|  | 4.000×10^4^ | 11.6 | 1.1 | 13.1 |
| L-arginine | 1000 | 2.3 | 2.1 | 2.1 |
|  | 1.000×10^4^ | 5.0 | 5.2 | -12.7 |
|  | 4.000×10^4^ | 6.2 | 6.4 | -12.3 |
| S-adenosyl-L-methionine | 4.00 | 10.3 | 11.6 | -13.6 |
|  | 40.00 | 3.5 | 0.2 | 11.1 |
|  | 160.0 | 4.1 | 4.4 | 1.8 |
| N-acetylputrescine | 0.20 | 9.1 | 7.3 | 4.34 |
|  | 2.00 | 8.2 | 0.8 | 10.4 |
|  | 8.00 | 6.5 | 9.6 | 9.6 |
| N-acetylspermine | 0.20 | 14.6 | 3.4 | 2.0 |
|  | 2.00 | 9.6 | 4.5 | 11.4 |
|  | 8.00 | 2.6 | 13.1 | -3.9 |
| N-acetylspermidine | 0.20 | 13.1 | 6.6 | -10.9 |
|  | 2.00 | 4.2 | 4.8 | -5.5 |
|  | 8.00 | 6.0 | 7.1 | 5.6 |
| γ-aminobutyric acid | 100.0 | 3.2 | 1.5 | -6.1 |
|  | 1000 | 4.4 | 13.9 | 13.4 |
|  | 4000 | 13.7 | 14.2 | -8.9 |

The method validation of precision and accuracy for the analytes in plasma (*n*=6)

The method validation of recovery for the analytes in plasma (*n*=6).

| Analytes | Recovery (%) | | |  | RSD (%) | | |
| --- | --- | --- | --- | --- | --- | --- | --- |
|  | Low | Medium | High |  | Low | Medium | High |
| 1,3-diaminopropane | 73.77 | 67.79 | 73.62 |  | 3.4 | 3.5 | 5.0 |
| putrescine | 63.67 | 60.79 | 78.58 |  | 6.6 | 6.2 | 7.2 |
| cadaverine | 62.78 | 69.62 | 77.64 |  | 4.8 | 5.4 | 5.1 |
| spermidine | 78.84 | 76.83 | 74.78 |  | 1.8 | 1.5 | 1.8 |
| spermine | 71.75 | 70.85 | 66.75 |  | 3.8 | 4.2 | 3.1 |
| agmatine | 68.55 | 77.69 | 60.71 |  | 7.5 | 7.2 | 7.2 |
| L-ornithine | 69.81 | 67.55 | 67.75 |  | 12.9 | 12.1 | 11.1 |
| lysine | 65.58 | 77.74 | 62.82 |  | 7.5 | 10.5 | 9.7 |
| L-arginine | 79.71 | 70.62 | 63.66 |  | 4.7 | 5.3 | 3.9 |
| S-adenosyl-L-methionine | 67.71 | 79.81 | 66.67 |  | 1.1 | 1.9 | 1.4 |
| N-acetylputrescine | 71.57 | 78.79 | 60.74 |  | 8.2 | 8.8 | 7.4 |
| N-acetylspermine | 62.61 | 67.67 | 72.83 |  | 9.9 | 9.1 | 8.8 |
| N-acetylspermidine | 71.62 | 74.74 | 62.59 |  | 14.6 | 14.4 | 14.4 |
| γ-aminobutyric acid | 78.58 | 64.68 | 79.58 |  | 2.7 | 2.6 | 1.3 |

| Analytes |  | IS-normalized matrix effect (%) | | |
| --- | --- | --- | --- | --- |
|  |  | Low | Medium | High |
| 1,3-diaminopropane |  | 2.4 | 8.5 | 14.2 |
| putrescine |  | 8.9 | 6.2 | 10.8 |
| cadaverine |  | 8.0 | 5.3 | 9.2 |
| spermidine |  | 4.6 | 3.1 | 5.4 |
| spermine |  | 1.5 | 8.0 | 13.5 |
| agmatine |  | 9.4 | 5.2 | 8.7 |
| L-ornithine |  | 12.6 | 11.1 | 9.8 |
| lysine |  | 6.4 | 5.5 | 9.9 |
| L-arginine |  | 12.1 | 12.1 | 1.9 |
| S-adenosyl-L-methionine |  | 3.4 | 9.7 | 6.2 |
| N-acetylputrescine |  | 2.3 | 1.4 | 2.5 |
| N-acetylspermine |  | 4.2 | 0.9 | 1.0 |
| N-acetylspermidine |  | 8.1 | 4.8 | 8.0 |
| γ-aminobutyric acid |  | 9.8 | 7.4 | 12.9 |

The method validation of matrix effect for the analytes in plasma (*n*=6).

The method validation of stability for the analytes in plasma (*n*=6).

| Analytes | Stability-long (at -80 °C for 1 month) | | Freeze-thaw-3 ( Freeze at-80 °C for 12h, thaw at 4 °C for 12 h) | | Stability- 4h (at room temperature for 4 h) | | Autosampler-12h (at 4 °C for 12 h) | |
| --- | --- | --- | --- | --- | --- | --- | --- | --- |
|  | Low | High | Low | High | Low | High | Low | High |
| 1,3-diaminopropane | 14.0 | 11.5 | 10.4 | 1.1 | 1.2 | 3.3 | 12.2 | 12.6 |
| putrescine | -12.6 | -14.5 | 5.2 | -5.2 | 6.4 | -12.5 | -11.6 | -12.8 |
| cadaverine | 11.5 | 14.1 | 6.3 | 6.9 | 7.8 | 13.3 | 13.2 | 13.6 |
| spermidine | 9.2 | 8.3 | 1.4 | 12.6 | 13.1 | 7.7 | -6.1 | 7.2 |
| spermine | 10.5 | -11.3 | 8.8 | -6.5 | 7.9 | -14.5 | 4.1 | -4.2 |
| agmatine | 13.8 | 11.3 | 2.2 | 7.8 | 7.3 | 10.6 | 3.2 | 3.4 |
| L-ornithine | 10.0 | 7.0 | 7.2 | 14.6 | 11.9 | 3.4 | 1.3 | 2.6 |
| lysine | -9.5 | 5.0 | -8.5 | 6.5 | -7.3 | 13.6 | -6.5 | 8.0 |
| L-arginine | 12.7 | 10.5 | 8.0 | 13.0 | 12.4 | 2.1 | 10.2 | 11.5 |
| S-adenosyl-L-methionine | 10.1 | 9.8 | 7.8 | 2.7 | 3.4 | 6.2 | 4.0 | 4.1 |
| N-acetylputrescine | 12.4 | -8.5 | 4.5 | -12.8 | 13.6 | -6.4 | 3.4 | -4.9 |
| N-acetylspermine | 3.3 | 0.5 | 1.0 | 9.9 | 10.1 | 9.8 | 2.6 | 3.3 |
| N-acetylspermidine | -3.2 | 0.3 | -1.9 | 14.1 | -10.0 | 5.5 | -10.7 | 11.6 |
| γ-aminobutyric acid | 7.5 | 3.3 | 5.6 | 8.9 | 9.9 | 7.9 | 9.2 | 9.4 |
